# Patients’ and Parents’ Perceptions of a “Good” Orthodontic Treatment

**DOI:** 10.7759/cureus.89295

**Published:** 2025-08-03

**Authors:** Nikolaos Ferlias, Cecilie Krogsgaard, Signe Schaldemose, Ida Thousgaard Ohlsend, Peter Stoustrup

**Affiliations:** 1 Section of Orthodontics, Department of Dentistry and Oral Health, Aarhus University, Aarhus, DNK

**Keywords:** evidence-based orthodontics, orthodontic treatments, parental perception, patient-reported outcome, patients perception

## Abstract

Objective

This study aims to investigate (1) the factors that pediatric orthodontic patients consider important in defining a *good* orthodontist and treatment process, and (2) the core elements of a *good* orthodontic treatment as identified by young patients and their parents.

Methodology

Children and adolescent orthodontic patients were prospectively interviewed, along with a parent and their treating orthodontist, at Aarhus University’s postgraduate clinic, Denmark, using a reconstructive grounded theory approach. The study consisted of (1) four open-ended questions for patients, grouped into thematic domains, and (2) a closed-ended questionnaire, based on the World Health Organization’s pediatric healthcare quality framework, answered by patients and parents. Seven treatment-related items were rated on a Likert scale (1 = Not important at all to 5 = Very important).

Results

Thirty-six patients (mean age 13.7 years, standard deviation (SD) 1.9 years) and their parents participated. Most of the open-ended qualitative responses of a *good* orthodontic treatment referred to the following subdomains: *Communication* (52, 30%), *Information at the right time* (43, 25%), *Pain during treatment* (21, 12%), *Competence* (19, 11%), and *Respect and mutual trust* (18, 10%). In the closed-ended questionnaire, parents rate it with higher importance than the patients that the orthodontist is an expert (*P *= 0.01).

Conclusions

Young orthodontic patients consider the entire treatment process when evaluating a *good treatment* and a *good orthodontist*. The two main factors they value most are having a friendly orthodontist and being provided with valuable information on their orthodontic treatment at the right time. The findings of the current project can be used to improve the overall quality of care and increase patient satisfaction.

## Introduction

Historically, healthcare adopted an authoritative, paternalistic approach, long considered the norm in care [1]. However, technological advancements and widespread information access have empowered patients to take a more active role in the patient-doctor relationship. Today, they actively participate in health decisions, becoming partners in treatment [2]. Consequently, patient satisfaction and feedback have gained importance, making provider evaluation increasingly crucial [3].

Perceptions of *good* healthcare or providers are closely tied to patient expectations and beliefs. Patients assess treatment quality by comparing expectations with actual experiences [4]. While providers focus on outcomes, patients value the entire process [5]. Although ideal expectations are fixed, predicted expectations, shaped largely by provider behavior, can be influenced [6]. Past experiences, social interactions, and age also affect expectations and motivation for treatment [7].

Understanding patient perception and expectations is essential for improving health care quality. The World Health Organization (WHO) has proposed standards to improve pediatric healthcare, recognizing children’s unique physical, psychosocial, and developmental needs [8]. These standards reflect different domains, from evidence-based care to effective communication, emotional support, and can be integrated into orthodontic research [8].

Despite the growing focus on patient-centered orthodontic care, comprehensive studies exploring patient perceptions remain limited. Most literature focuses on clinical outcomes like occlusal improvement without exploring patient perspectives [9]. Additionally, studies exploring oral health-related quality of life overlook key factors shaping patients’ treatment experiences [10].

Inspired by the aforementioned WHO’s framework, this mixed-method study intends to expand knowledge on orthodontic patient perceptions. The aims are, first, to investigate the factors that children and adolescent patients consider important in a *good* orthodontist and treatment process, and second, to identify central issues in orthodontic care.

## Materials and methods

Data collection occurred at the postgraduate clinic at the Section of Orthodontics, Department of Dentistry and Oral Health, Aarhus University, Denmark, from September to November 2022, following methodological elements from reconstructing grounded theory where data collection and data analysis interwind [11]. Inclusion criteria were children undergoing orthodontic treatment with upper and lower full fixed orthodontic appliances over the age of 11 years, with a parent present at the time of interview. All interviews were conducted on consecutive patients who were invited to participate in the study, in a predefined manner by three of the authors (CK, SS, ITO) alongside a parent and the treating postgraduate student. Exclusion criteria were non-consenting patients and parents, and those unable to communicate effectively in Danish. Interviews were recorded, transcribed, and numerically coded to ensure confidentiality. Written informed consent was obtained from all participants (patients below 15 years of age consented and their parents signed and consented). 

The interviews followed a standardized script with five open-ended and seven closed-ended questions (Table 1; Appendices A-B), based on WHO’s framework and incorporating elements from the seven pillars of clinical governance: (1) Evidence-based care and effectiveness, (2) Clinical quality requirements, (3) Risk management, (4) Education and professional development, (5) Patient/public involvement, (6) Information management, and (7) Staff management [8,12]. Patients were first asked to confirm their age, gender, treatment stage, and motivation for treatment, which was rated on a 1-5 Likert scale (1= No motivation and 5 = Excellent motivation). 

**Table 1:** Open- and closed-ended questions. After the introductory questions (age, gender, motivation, etc.), patients were asked four open-ended questions (Q1-Q4) and were then given seven closed-ended questions (Q5-Q11) where they rated the importance of various aspects related to their orthodontic treatment. These closed-ended questions were also answered by their parents.

Questionnaire item	Question	Type of question	Assessment of outcome
Q1	What are the most important factors for you to feel that you are getting "really good" treatment?	Open-ended	Identifying domains and subdomains
Q2	What would it take for you to think that the treatment process is "really bad"?	Open-ended	Identifying domains and subdomains
Q3	Which of these things/conditions are the most important for you to think that the dentist is a "good" dentist?	Open-ended	Identifying domains and subdomains
Q4	Which of these things/conditions are needed for you to think that the dentist is a bad dentist?	Open-ended	Identifying domains and subdomains
Q5	Rate the importance: That your orthodontist is an "expert" in moving teeth and that your braces treatment is carried out according to the latest orthodontic guidelines.	Likert scale	1 = Not important at all, 2 = Not important, 3 = Both/and, 4 = Important, 5 = Very important
Q6	Rate the importance: Information about your treatment is shared/coordinated with your doctor/other dentists who also see you	Likert scale	1 = Not important at all, 2 = Not important, 3 = Both/and, 4 = Important, 5 = Very important
Q7	Rate the importance: That you do not have to wait to start treatment (no waiting list)	Likert scale	1 = Not important at all, 2 = Not important, 3 = Both/and, 4 = Important, 5 = Very important
Q8	Rate the importance: Before and during treatment, you receive information about your treatment and what to expect	Likert scale	1 = Not important at all, 2 = Not important, 3 = Both/and, 4 = Important, 5 = Very important
Q9	Rate the importance: That your orthodontist is friendly and approachable and supports you throughout your treatment	Likert scale	1 = Not important at all, 2 = Not important, 3 = Both/and, 4 = Important, 5 = Very important
Q10	Rate the importance: That you can easily get in touch with your orthodontist	Likert scale	1 = Not important at all, 2 = Not important, 3 = Both/and, 4 = Important, 5 = Very important
Q11	Rate the importance: That the equipment and the clinic are modern and new	Likert scale	1 = Not important at all, 2 = Not important, 3 = Both/and, 4 = Important, 5 = Very important
Q12	Which of the areas/conditions/questions you have been asked above are the most important for you to have a good course of treatment with braces?	Open-ended	The patient picks the most important theme.

A qualitative, inductive approach informed by grounded theory was applied to explore how patients and parents define a *good orthodontic treatment*. Free-text feedback was collected from patients and their parents, and responses were analyzed using a grounded theory framework to identify key themes emerging from the data. The analysis followed three iterative stages: (1) Open coding, where each response was reviewed line-by-line and coded to capture distinct concepts and perspectives expressed by the participants. (2) Axial coding, in which related codes were grouped to form broader thematic categories, highlighting relationships between experiences and perceived treatment quality. (3) Selective coding, where central domains and subdomains were defined to integrate the findings into a conceptual framework describing the core dimensions of positive treatment experience from the patient and parent perspective. The sample size was based on thematic saturation.

Coding was conducted manually, and themes were refined through constant comparison across responses, with discrepancies being discussed and resolved through consensus. The resulting conceptual model comprises overarching domains and subdomains that reflect the multi-dimensional nature of what constitutes *good orthodontic treatment* as perceived by patients and their families.

Statistics

Descriptive statistics were used for the data, and normality was assessed with the Shapiro-Wilk test. The Mann-Whitney U Rank test was used to evaluate the results from the closed-ended questions and compare children’s responses to their parents, using Excel® (Microsoft®, Redmond, WA).

Ethical approval

The study was conducted in agreement with the Danish Research legislation on questionnaire-based research. The project was approved by the Danish data protection authorities through the Aarhus University reporting scheme (2022-0367531, serial no. 3686). Informed consent was obtained from all eligible patients and their parents before inclusion in the study.

## Results

Thirty-six pediatric orthodontic patients and their parents were interviewed (17 boys and 19 girls). The mean age of the patients was 13.7 years (SD: 1.9 years, range: 11-18 years). The majority (*n* = 20, 56%) were 6-12 months into their orthodontic treatment, while 13 (36%) were already having treatment for 18 months or more. Three patients (8%) were at the start of their treatment. Most of these patients (23, 64%) were *very* or *highly* motivated for their orthodontic treatment.

Open-ended questions

Responses to the open-ended questions were grouped into three main domains based on their content, with answers potentially reflecting multiple domains, following inspiration from previous healthcare-related qualitative research (Table 2) [13]. These were (1) *Professional attitude of healthcare staff* (135, 78%), (2) *Quality of care delivery* (12, 7%), and (3) *Orthodontic treatment and result* (27, 15%). Answers belonging to the first domain, *Professional attitude of healthcare staff* (135, 78%), were further subdivided into the following five subdomains: (1A) *Respect and mutual trust* (18, 10%) (patient and orthodontist having mutual respect and engaging in a trusting relationship), (1B) *Information at right time* (43, 25%) (the orthodontist providing valuable information to the patient regarding their treatment, e.g., what to expect or what’s about to happen, why, etc.), (1C) *Shared decision-making* (when the orthodontist actively engages the patient in treatment-related decisions) (3, 2%), (1D) *Competence* (19, 11%) (when the patient feels the orthodontist is competent enough to deliver the treatment), and (1E) *Communication* (52, 30%) (when the orthodontist has good social skills, engages in conversation with patients/parents, and is pleasant to talk to).

**Table 2:** Domains and subdomains from the patients' answers: Clusters and frequency of the open-ended responses in three domains (“Professional attitude of healthcare staff,” “Quality of care delivery,” and “Orthodontic treatment and end result”) and their subdomains.

Domain	Professional attitude of healthcare staff (*n *= 135)		Quality of care delivery (*n *= 12)		Orthodontic treatment and end result (*n *= 27)	
Subdomains	Communication	52	Facilities	5	Pain during treatment	21
	Information at the right time	43	Accommodation	2	Quality of end result	4
	Competence	19	Continuity of care	2	Duration of treatment	2
	Respect and mutual trust	18	On-time delivery of care	2		
	Shared decision-making	3	Access to care	1		

The second domain, *Quality of care delivery* (12, 7%), consisted of the following subdomains: (2A) *Access to care* (how easy it is for someone to get orthodontic treatment) (1, 0.5%), (2B) *Accommodation *(2, 1%) (appointment availability, accessibility issues, ease at accommodating patients’ requests during delivery of care), (2C) *Continuity of care *(2, 1%) (patients seeing the same clinician on every appointment, no unpleasant surprises during treatment), (2D) *Facilities *(5, 3%) (orthodontic clinic facilities, cleanliness, quality of equipment, etc.), and (2E) *On-time delivery of care *( 2, 1%) (minimum waiting time at appointments).

The final and third domain, *Orthodontic treatment and quality of end result *(27, 15%) consisted of the following subdomains: (3A) *Pain during treatment* (21, 12%) (when patients experience pain during their treatment or when the orthodontist is a bit *rough* during their appointments and hurts them unnecessarily), (3B) *Quality of end result* (4, 2%) (how good is the final result of the orthodontic treatment in terms of occlusion and dental aesthetics through the patient’s eyes), (3C) *Duration of treatment *(2, 1%) (when the treatment takes longer that initially agreed or expected).

Most patients' responses fell under the domain *Professional attitude of healthcare staff* (135, 78%), with results summarized in Table 2. The most cited subdomains were *Communication* (52, 30%), and *Information at the right time* (43, 25%), highlighting the importance of a friendly orthodontist and clear information during treatment. These were followed by *Competence* (19, 11%) and *Respect and mutual trust* (18, 10%). Additionally, *Pain during treatment* (in the *Orthodontic treatment and end result* domain) was also frequently noted (21, 12%). Examples of answers categorized by domains and subdomains are shown in Table 3.

**Table 3:** Examples of responses to the four open-ended questions with their domains and subdomains. Q1: “What are the most important factors for you to think you are getting a "really good" treatment?” Q2: “What would it take for you to think the treatment process is "really bad"?” Q3: “Which of these things/conditions are the most important for you to think the dentist is a "good" dentist?” Q4: “Which of these things/conditions would make you think the dentist is a bad dentist?” Some replies referred to more than one domain/subdomain. For example, patient in Q1 is referring to both good communication with the orthodontist as well as receiving the right information on what to expect during treatment (*Professional attitude of healthcare staff* domain). Patient in Q2 appreciates the importance of cleanliness in the clinic as well as having no waiting time (*Quality of care* domain).

Open-ended question	Quotation	Domain	Subdomain
Q1	“Well, I would say it’s both the therapists, the ones you are treated by. Yes, there are many things that go into it, it can be something like how you are spoken to, and how much information you get and, yes, the communication.”	Professional attitude of healthcare staff	Information at the right time and Communication
Q1	“Cleanliness and tidiness, and that times are convenient so that you don’t spend too long in the waiting room.”	Quality of care delivery	Facilities and on-time delivery of care
Q2	“That it does not hurt.”	Orthodontic treatment and end result	Pain during treatment
Q2	“Mmmmh it’s a bit difficult to answer, but probably if it’s something you are not pressured to do and if they just do something you were not aware of and do not give information about what they are doing.”	Professional attitude of healthcare staff	Information at the right time
Q2	“If you are not explained what is happening, a rude dentist.”	Professional attitude of healthcare staff	Information at the right time and communication
Q3	“That the dentist says what they do and doesn’t just expect you to know.”	Professional attitude of healthcare staff	Information at the right time
Q4	“If you are not satisfied with the work, I would say, the result. Or you feel that you have not been treated as you should have been.”	Orthodontic treatment and end result	Quality of end result
Q4	“If the dentist is dictatorial and does not speak nicely.”	Professional attitude of healthcare staff	Shared decision-making and communication

Closed-ended questions

**Figure 1:**
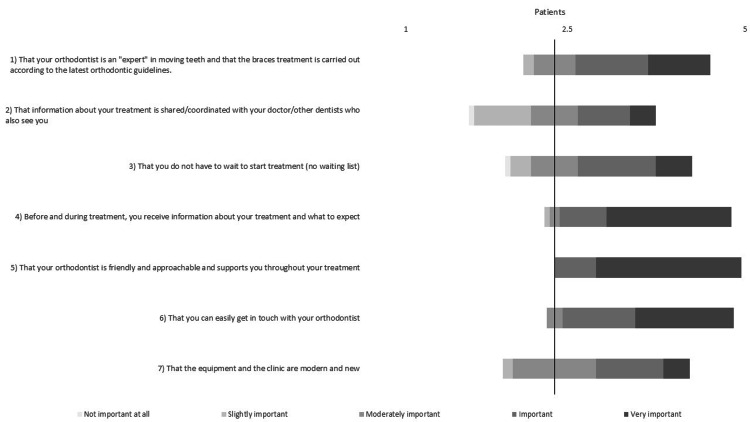
Bar chart based on patients’ responses to the Likert-format, closed-ended questions (Likert scale range: 1-5; 1 = Not important, 2 = Slightly important, 3 = Moderately important, 4 = Important, 5 = Very important).

**Figure 2:**
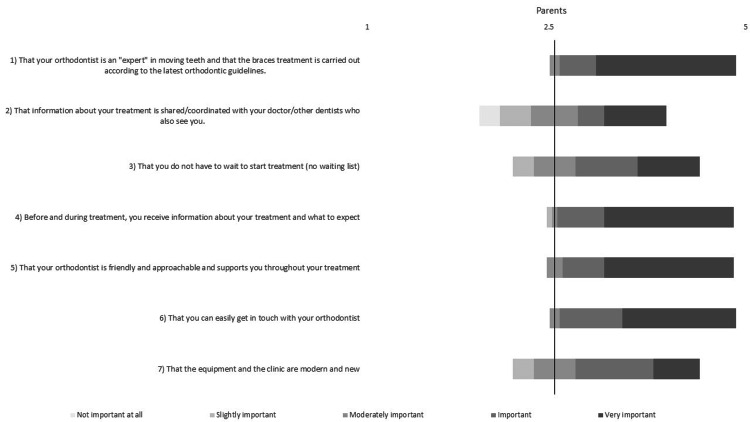
Bar chart based on parents’ responses to the Likert-format, closed-ended questions (Likert scale range: 1-5; 1 = Not important, 2 = Slightly important, 3 = Moderately important, 4 = Important, 5 = Very important).

The closed-ended questions were answered by both patients and parents (Figures 1-2). The Shapiro-Wilk test revealed non-normal data distribution; therefore, the non-parametric Mann-Whitney U rank test was used. Both parents’ and patients’ answers followed a similar pattern, indicating that they both consider similar topics important. The three highest-rated themes for the patients were: (1) having a friendly and approachable orthodontist who supports them throughout treatment (Likert score ± SD: 4.78 ± 0.42); (2) receiving information about the treatment and what to expect (Likert score ± SD: 4.56 ± 0.73); and (3) being able to easily get in touch with the orthodontist (Likert score ± SD: 4.44 ± 0.65). The three highest-rated themes for the parents were: (1) that the orthodontist is an *expert* who follows the latest guidelines (Likert score ± SD: 4.69 ± 0.58); (2) receiving information about the treatment and what to expect (Likert score ± SD: 4.61 ± 0.69); and (3) having a friendly and approachable orthodontist who supports the patient throughout treatment (Likert score ± SD: 4.61 ± 0.64).

Apart from question 1 (Q1), no significant differences were found between children and parents regarding the importance of receiving treatment information, starting treatment promptly, having a friendly orthodontist who is approachable and easy to get in touch with, and a clinic that is modern and new (*P* > 0.05). However, parents rated the importance of an expert orthodontist carrying out the treatment according to the latest guidelines significantly higher than the patients (*P* = 0.01).

**Figure 3:**
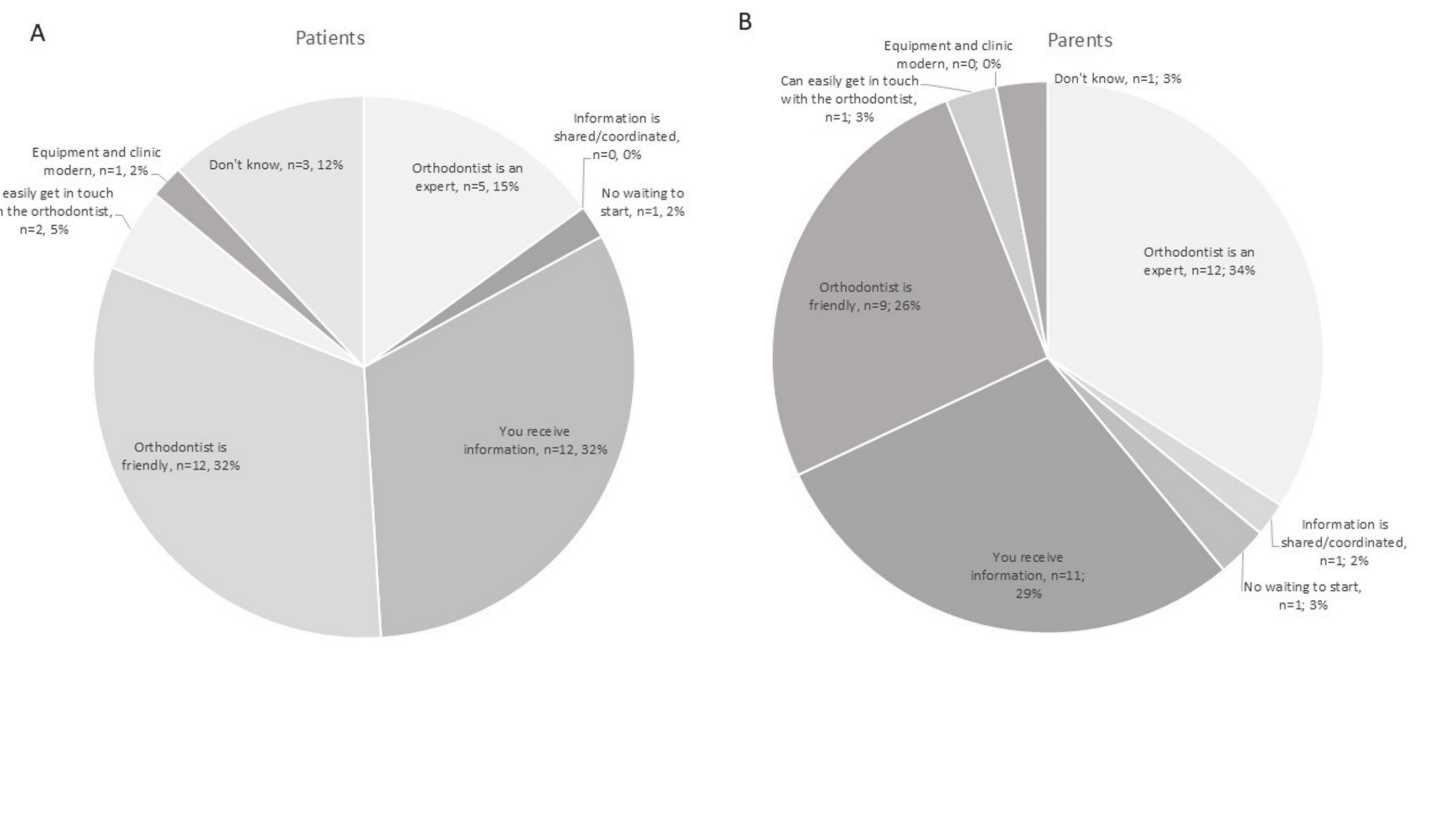
Results from patients’ (A) and parents’ (B) replies to the open-ended final question, Q12: Which of the areas/conditions/questions you have been asked above are the most important for you to have a good course of treatment with braces? The areas refer to the closed-ended questions (Q5-Q11).

On the final (and open-ended) question (Q12), patients and parents were asked to rate the importance of the aforementioned closed-ended questions (Figure 3). Most patients considered having a friendly orthodontist (32%) and receiving treatment information (32%) the most important areas in their evaluation. Similarly, parents rated these highly (26% and 29%, respectively) but considered having an expert orthodontist most important (34%), highlighting a key difference in priorities.

## Discussion

This mixed-methods study evaluated patients’ and parents’ perceptions of orthodontic treatment, exploring what defines a *good* orthodontist using both quantitative and qualitative questions aligned with WHO’s standards for pediatric care. Results highlight the importance of positive behavioral traits, particularly communication skills and timely, useful information, in shaping patients’ views. Additionally, findings reveal that while parents and young patients share similar perceptions of a *good* orthodontic treatment, parents place greater emphasis on the orthodontist's expertise.

Previous studies have shown that communication is considered vital in the doctor-patient relationship [14], [15-17]. Clayton et al. defined patient-centered communication as “care that is respectful of and responsive to individual patient preferences, needs, and values and that demonstrates partnering skills and relationship building” [18]. The results of our study come in agreement with similar investigations on the importance of communication on what constitutes a *good* doctor, where attributes like *friendly*, *funny*, and *sociable* were used by young patients to describe a *good* doctor [19]. Furthermore, communication acts as the initial pillar on which a trusting doctor-patient relationship is built. In addition, since the attributes within the subdomains used in this study to group the responses of our cohort are intertwined and overlapping, it is evident that one attribute can lead to another. For example, good communication is important in order to build respect and mutual trust as well as provide valuable information to our patients.

Furthermore, researching patient-reported outcomes and experiences, as well as the aforementioned communication and information, is the basis of clinical governance in patient and public involvement [12,20]. Our results show that patients value receiving timely, valuable information, whether at the start of treatment or about upcoming steps. This fosters security, reduces anxiety, aligns expectations, and minimizes unpleasant surprises. Additionally, it plays a key role in obtaining informed consent. At the start of orthodontic treatment, patients and orthodontists must navigate complex decisions involving various options, treatment plans, and appliances. Our findings align with previous research emphasizing the importance of communication during this critical phase [21].

Moreover, clinical competence is considered necessary in improving the delivery of care according to WHO’s standards for improving the quality of care [8]. Our results support this statement, from the patient’s perspective, as the orthodontist’s competence was deemed important. This is vital because it enhances a trusting and respectful doctor-patient relationship [22]. It also boosts patients’ confidence in the treatment and can potentially lead to better compliance and treatment outcomes [23]. A positive doctor-patient relationship contributes to higher patient satisfaction levels [5]. Nevertheless, the orthodontist’s competence is difficult to quantify and evaluate from the patient’s perspective due to a lack of technical knowledge on their part. Since they find it difficult to critically assess it, they tend to interpret the expression of confidence as competence [6]. Therefore, our results support the notion that the orthodontist’s interpersonal skills seem to be central in this process.

Pain management is a key factor in enhancing compliance and satisfaction [24]. Many open-ended responses highlighted the importance of providing information about *pain* and *discomfort* beforehand. Our results show this is a key concern for pediatric orthodontic patients. As expected, pain triggers negative emotions, including anxiety, fear, and uncertainty about what lies ahead [25]. Effective pain management is achieved through appropriate communication and the dissemination of accurate information before and during treatment. Evidence suggests that even a follow-up text or phone call after placing an orthodontic fixed appliance can significantly improve patients' perceptions of pain and its impact on daily life [26,27].

Data from the closed-ended questions reveal a similar pattern in patients’ perceptions. Key valued elements include being treated by an expert who also provides valuable information, and most importantly, an orthodontist who is friendly and approachable. This highlights the importance of social and communication skills, both verbal and non-verbal, beginning with the first impression during the initial consultation. These findings align with previous investigations showing that humanistic traits like emotional sensitivity and positive personality often define a “good doctor” more than scientific proficiency [14]. 

The statistical significance in closed-ended question 5 (Appendix A) shows that parents place significantly greater importance on the orthodontist’s expertise compared to their children undergoing treatment. We hypothesize that it probably has to do with the difference in cognitive and emotional maturity between parents and children. However, in the two highest-scoring items, both parents and children seemed to agree on the level of importance. This was the information they received before and during their treatment, and the friendliness of the orthodontist. Pediatric patients appreciate having valuable information regarding their treatment, and this helps them feel more secure and confident as they know what to expect. This minimizes any unpleasant surprises, encourages compliance, and enhances the overall experience.

Limitations/Strengths

This study comes with limitations. Patients’ responses may have been influenced by the presence of a parent or treating doctor. Children and adolescents might alter their feedback - consciously or unconsciously - to align with perceived expectations or to avoid potential judgment when authority figures are present. In addition, self-report bias and social desirability effects might also have affected the responses. They might have also been influenced by the fact that almost all patients were receiving tax-funded, free orthodontic treatment (only two paid for their treatment). Additionally, the generalizability of these findings should be done with caution, as these may be more relevant to Denmark due to cultural differences in how patients receive orthodontic treatment from the state, or even cultural differences. Future multicenter, intercultural studies could further assess the generalizability of these results. Finally, as with all questionnaire-based studies, there is a risk of patients providing overly positive evaluations due to psychosocial barriers [28].

Important strengths of the present study are the fact that this is the first patient-centered research attempt to investigate patients’ perception and experience of the whole orthodontic treatment process through the WHO’s standards for improving quality of care [8]. For this reason, the questionnaire used was formulated to include information on these standards with an interview that was standardized and structured. Therefore, data was collected through an interview process rather than just the completion of a questionnaire. Another strength is that the interview technique was based on principles of *reconstructing grounded theory*, which is a more dynamic process compared to other qualitative observations that are static [11]. The main advantage of grounded theory is that data collection and analysis intertwine, and new insights constantly change and optimize our understanding as we dig deeper to find more information during the data collection process. Another strength is that the timing of treatment was different from patient to patient, which gave a better insight into all facets of the orthodontic treatment process.

The present study is considered a *pilot project*, with results intended to guide future work in this important area of quality assurance in orthodontics.

Clinical implications

Our findings highlight key areas that can be addressed regarding quality assurance and patient satisfaction assessments. Additionally, these results prompt reflection on how we design clinical patient pathways. Achieving high levels of patient satisfaction is not solely dependent on professional expertise but requires a respectful and positive patient-doctor relationship, where communication plays a crucial role. Future studies, though multicenter, longitudinal, and culturally diverse settings, should investigate potential intercultural differences that may influence this dynamic. This will help assess generalizability but also perceptions' evolution across treatment stages with follow-up studies.

## Conclusions

Young patients have a *holistic* view of various aspects of the treatment process when evaluating a *good treatment* and a *good orthodontist*. The two main factors they value most are good communication with the orthodontist and being provided with valuable information on their orthodontic treatment at the right time.

Perceptions of orthodontic patients and their parents on factors affecting orthodontic treatment follow a similar pattern. Both patients and parents of patients value having a friendly and approachable orthodontist and receiving important information on what to expect. However, parents appreciate much more the importance of having an orthodontist who is an expert. The findings of the current project can be used to improve the overall quality of care and increase patient satisfaction.

## Appendices

Appendix A 

**Table 4:** Interview script that was used in all interviews.

Please rate the importance of each statement (1= not at all important), 5= very important)	
Question	
1) That your orthodontist is an "expert" in moving teeth and that the orthodontic treatment is carried out according to the latest orthodontic guidelines.	□ 1) Not important at all □ 2) not important □ 3) both/and □ 4) Important □ 5) Very important
2) That information about your treatment is shared/coordinated with your doctor/other dentists who also see you	□ 1) Not important at all □ 2) not important □ 3) both/and □ 4) Important □ 5) Very important
3) That you do not have to wait to start treatment (no waiting list)	□ 1) Not important at all □ 2) not important □ 3) both/and □ 4) Important □ 5) Very important
4) Before and during treatment, you receive information about your treatment and what to expect	□ 1) Not important at all □ 2) not important □ 3) both/and □ 4) Important □ 5) Very important
5) That your orthodontist is friendly and approachable and supports you throughout your treatment	□ 1) Not important at all □ 2) not important □ 3) both/and □ 4) Important □ 5) Very important
6) That you can easily get in touch with your orthodontist	□ 1) Not important at all □ 2) not important □ 3) both/and □ 4) Important □ 5) Very important
7) That the equipment and the clinic are modern and new	□ 1) Not important at all □ 2) not important □ 3) both/and □ 4) Important □ 5) Very important
8) Which of the areas/conditions/issues you have been asked about above are the most important for YOU to have a good course of treatment with braces?	

Appendix B 

**Table 5:** All patients’ answers to the four open-ended questions, Q1-Q4.

Patient	Q1: What are the most important factors for you to think you are getting a "really good" treatment?
1	If the practitioner is nice and friendly and you feel comfortable.
2	I don't know, I don't really think there is
3	Well, someone who's good at that kind of braces thing.
4	That the dentist is good
5	It's the staff, they have to be nice
6	I don't know, I think it's really cool when they are nice and you can see them laughing and you have a little fun with it
7	That the people treating me are nice, and in a good mood, and it's clean, just generally a good environment
8	That I also have a say in how it is done, and that it is done properly, instead of quantity then quality.
9	That I have a really nice dentist who is careful with his mouth so he doesn't do anything that hurts.
10	It's really important to me that my teeth stay in place, because I wouldn't go through all this just because it doesn't work.
11	Well, I would say it's both the therapists, the ones you are treated by. Yes, there are many things that go into it, it can be something like how you are spoken to, and how much information you get and, yes, the communication.
12	That they do it well (the result ends well)
13	The dentists, that they are nice
14	I am happy that my dentist tells us what to do before she does something and explains so that the patient can understand
15	That's very nice. That it's a nice dentist
16	Good atmosphere, lots of information about what's going to happen, nice waiting room
17	That it is not uncomfortable to be here. Not too formal, preferably personal
18	Explains what is going to happen beforehand, so you know what is going on
19	cleanliness and tidiness, and that times are convenient so that you don't spend too long in the waiting room
20	It is important to have a good connection with your dentist when you have them for so long
21	Proper communication about what is going to happen
22	Good communication, explaining what needs to be done, not just putting your fingers in your mouth
23	People are good
24	Dentists speak loud and clear otherwise everything is fine
25	Someone who is good at explaining things in good language, easy to talk to, approachable and open
26	To get as little damage as possible inside the mouth. In other words, that it hurts as little as possible.
27	The people doing the treatment are good at explaining it and that you know what you are getting into
28	That it does not hurt
29	That the dentist is nice and kind
30	That it does not hurt and that there is no stress when you are in the chair
31	Don't know, generally satisfied going to the dentist
32	Finishing on time and coming in for treatment on time
33	That there is a good view (window seat), that you have the same therapist/supervisor for each treatment
34	Having a problem that is being fixed
35	A good practitioner who explains what they do during the treatment
36	Good surroundings and that the people at the clinic are nice
Patient	Q2: What would it take for you to think the treatment process is "really bad"
1	Mmmmh it's a bit difficult to answer, but probably if it's something you are not pressured to do and if they just do something you were not aware of and do not give information about what they are doing.
2	It sucks when your mouth gets all dry, otherwise it's fine
3	I've had my lip torn and it split, and it wasn't fun
4	No, I've never thought about that, as long as they are good at it
5	I don't know about that.... the dentist is bad to you
6	Something that makes me uncomfortable, no I don't think so
7	I don't know, if it's just going slowly and you're just there. If they do it too fast, because then it can hurt a bit
8	if you had a lot of bad experiences, like if you asked for something and it didn't happen, and if it wasn't under control
9	If I have had a bad experience, if there is something that is unpleasant, or they don't listen or you have a say in the matter.
10	If he just pulls my cheeks, for example, really hard and, for example, comes to pinch me with scissors, or he just doesn't think about what he's doing and just tries to get it done quickly.
11	I don't want to go in for a treatment and just think it's the same thing that always happens, and then suddenly something happens, like I've got rubber bands and all sorts of things. It happened today, but I was told beforehand that this was what was going to happen
12	It's probably the opposite of miscommunication and yes, and if it feels unsafe, but it can also be one of the things miscommunication can lead to. What is meant by unsafe? - an example is asked about if the therapist does not seem qualified, here the pt answers yes.
13	If it ends up looking ugly or if they speak badly
14	That they are angry or if it hurts a lot (it's okay if it hurts a little)
15	If they don't really talk to me or show me what they are doing
16	If she is not nice and just does what she has to do
17	If they don't take good care of your teeth, if they fiddle with things a lot
18	If someone doesn't think about how the patient is feeling, e.g. if you have a very painful tooth and the dentist just pulls it
19	Not explaining properly what is going to happen
20	If you are not explained what is happening, a rude dentist
21	If they don't care so much about whether it hurts
22	Poor communication if you are not told what is going to happen
23	The opposite, if I don't know what is going to happen
24	That it hurts
25	If they scratch hard and it hurts and they don't pay attention
26	A practitioner who doesn't give breaks, someone who doesn't ask questions, because some things hurt
27	When it hurts
28	Don't know
29	If it hurts and takes a long time
30	Don't know
31	If it hurts and if something needs to be changed because the treatment has not worked
32	If the treatment takes too long, if there is a lot to be done at once
33	Don't know
34	If you do not have the same practitioner/counsellor every time
35	That it does not hurt
36	Don't know
Patient	Q3: Which of these things/conditions are the most important for you to think the dentist is a "good" dentist
1	The same
2	That the dentist is nice, and kind of thinks about what you do on a not just scratch, but say are you ready for this
3	I don't know. but they can be cute
4	that they tell me how it's going to do, the brace, what it's going to do
5	That the dentist is nice and good at his work
6	dentist must be nice
7	That the person is happy and is fresh in a way and always wants to help and wants to answer questions and ask us questions
8	someone who is smiling, happy and always open
9	I don't really know. I actually don't think it has that much influence.
10	That he is nice to me, that he does everything he can to make me feel good.
11	A mixture, I would say, because the treatment is of course crucial, it's the one you kind of have to have, but it's also important with the way it's done. It can be something that contributes to whether you recommend a place, whether you feel you have been treated properly. Also because you pay for it.
12	Probably the way she talks and that it will be good.
13	That I know what I need to do
14	Talking to me and telling me what is going to happen
15	Be thorough and explain what is happening so that children understand
16	That she is skilled and tells you what is going to happen. It's nice to know that you no longer have to wear a bracket, for example
17	That they check on my well-being and are not annoying
18	Maybe they ask questions that are not just about teeth, e.g. are you having a good day?
19	Welcoming. Is good at taking into account when you can come
20	The way they talk to you, the way they treat you, e.g. if they are not so hard on you, they show a little consideration
21	How good they are but specifically that it does not hurt. Would rather be anesthetized so that it does not hurt e.g.
22	A good dentist is good at listening to what the patient needs, for example if the patient says no or it hurts. The dentist knows what is going on.
23	Very personable, good personality when they talk a lot and ask about other things.
24	They are nice, kind and do a good job
25	Someone who is not afraid to ask for help perhaps, and say they have made a mistake
26	That they listen when something hurts
27	That they explain the things to be done (information)
28	That the dentist says what they do and doesn't just expect you to know
29	That it does not hurt and that the dentist is good at explaining what is happening in the treatment
30	That the dentist is friendly, open to questions, good at telling what is going to happen
31	That the dentist is nice, is not too violent during the treatment, asks if you are OK and if it hurts
32	Involves the patient in what is going to happen/tells about the treatment
33	That the dentist is helpful and interested in the profession and the patient in the chair
34	That the dentist explains what is happening, that it does not hurt
35	That the dentist explains what they do during the treatment and is good at explaining
36	Don't know
Patient	Q4: Which of these things/conditions would make you think the dentist is a bad dentist?
1	It is also the same as before
2	Someone who isn't nice and doesn't tell you what's going to happen.
3	I don't know...
4	Actually, I don't think there is
5	If they treat you badly
6	yes if the dentist is not nice
7	Someone who just wants to get it over with and doesn't really want to talk and just ask the parents for something
8	someone who seems a bit tired and grumpy and not really bothered
9	I don't know, I find that a bit difficult.
10	That he doesn't care how I feel, that he just wants to get his work done without thinking about the patient
11	If you are not satisfied with the work, I would say, the result. Or you feel that you have not been treated as you should have been
12	If they speak badly
13	That they are unpleasant (e.g. angry) and I don't know what is happening
14	If she doesn't talk and doesn't tell us what to do. That she is completely silent
15	Not explain it so thoroughly
16	If she does not tell you what is happening
17	That they do not take into account
18	If they are really angry
19	All the opposite
20	If they don't say much, are in a hurry and just need to get it done
21	Poor communication
22	Opposite
23	Not talking so much and not being so nice
24	If they scratch hard and it hurts
25	Someone who does not explain and tell what is happening
26	If they do not listen to that it hurts
27	If the dentist does something without telling you they are doing it, you can get a shock
28	That the dentist says things in an angry way or that the dentist is angry
29	If the dentist does unpleasant things because it is difficult to stop while the practitioner is doing it.
30	If the dentist is angry/negative, does not say much and does not inform if something they are about to do might hurt
31	If the dentist is too violent or it hurts
32	If the dentist is too violent during treatment
33	If you can feel that the dentist does not know the subject and that the dentist is not nice/friendly
34	If the dentist is dictatorial and does not speak nicely
35	If the dentist does not inform about what they do in the treatment
36	Don't know
